# A Divide between the Western European and the Central and Eastern European Countries in the Peripheral Vascular Field: A Narrative Review of the Literature

**DOI:** 10.3390/jcm10163553

**Published:** 2021-08-12

**Authors:** Endre Kolossváry, Martin Björck, Christian-Alexander Behrendt

**Affiliations:** 1 Department of Angiology, St. Imre University Teaching Hospital, 1115 Budapest, Hungary; 2 Department of Surgical Sciences, Section of Vascular Surgery, Uppsala University, 75121 Uppsala, Sweden; martin.bjorck@surgsci.uu.se; 3 Department of Vascular Medicine, Research Group GermanVasc, University Medical Center Hamburg-Eppendorf, 20246 Hamburg, Germany; behrendt@hamburg.de

**Keywords:** health services research, regional differences, East–West divide, quality improvement, cardiovascular disease, outcomes research

## Abstract

Thirty years after the transition period, starting from 1989, Central and Eastern European countries (CEECs), representing one-fifth of the entire European population, share many historical, societal, political, economic, and cultural characteristics. Although accumulating data on coronary heart diseases and cerebrovascular diseases support these observations, in the case of peripheral arterial disease, data are scarce. The present review attempts to summarise the shreds of data that may highlight a divide in this field between CEECs and Western European countries. Disparities in risk factors and peripheral vascular care across Europe seem to be tangible and can be seen as a signal of existing differences. Improvements in research and development and the collection and cross-border share of scientific data are essential to initiate and facilitate convergence in this field.

## 1. Introduction

The history of the European Union (EU) since the 1957 signing of the Treaty of Rome by the six founding members (Belgium, France, Germany, Italy, Luxembourg, and the Netherlands), may be seen as waves of successful enlargements. From 1973 to 1995, nine other members joined (Denmark, Ireland, the United Kingdom, Greece, Portugal, Spain, Austria, Finland, and Sweden), forming the EU15 countries. After a long process of cooperation and preparation, an unpreceded accession took place with the integration of several countries from the Central and Eastern European Region. In the first wave of accession in 2004, eight states (Estonia, Latvia, Lithuania, Czech Republic, Slovakia, Poland, Hungary, and Slovenia) joined. Romania and Bulgaria followed them in 2007, and Croatia joined in 2013, leading the formation of EU13 countries, including Malta and Cyprus. The latter countries represent a different evolution compared with the former socialist states. Only fifteen years after the transition from a more or less totalitarian political regime, planned economy, and socialism towards a democratic regime, a market economy, and capitalism, these Central and Eastern European Countries (CEECs) were markedly challenged in many aspects of reshaping their system. The population of CEECs represents one-fifth of the entire population of the EU. In the 1990s, several studies had already indicated that this population exhibited poorer health and shorter life expectancy than the population of the Western European countries (WECs) [1]. This difference has been referred to as ‘The European Health Divide’.

Around the accession dates of the CEECs, data from this region suggested apparent disadvantage in many aspects of health, including life expectancy and mortality due to cancer or external causes. Rate of deaths due to cardiovascular disease (CVD), ischaemic heart disease, or cerebrovascular disease was also higher in CEECs than in WECs [2]. The amount of data on health status related to CVD in CEECs is increasing due to the participation of these countries in different population-based CVD registries. These registries, which include the Enhancing and Accelerating Stroke Treatment (ESO-EAST) collaborative project [3], the EUROASPIRE project [4], the REACH Registry [5,6], and the VASCUNET collaboration [7], support gathering data from CEECs. Additionally, an increasing number of isolated national reports from CEECs are being published, providing information about different domains of the vascular field.

In the present review, we seek to summarise data on a potential East–West divide across Europe in the peripheral vascular field by [1] assessing the epidemiological data related to atherosclerotic vascular risk and [2] exploring the differences in various aspects (structure, processes, outcomes) of peripheral vascular care (Table 1).

## 2. Methods

A comprehensive narrative electronic search was performed by the authors using the search engine PubMed (US National Library of Medicine) to access databases from MEDLINE, OLDMEDLINE, and PubMed Central. We used a combination of the following search terms, including appropriate synonyms: European health divide AND peripheral artery disease.

## 3. East–West Health Divide

### 3.1. East–West Disparity across Europe in the Pattern of Vascular Risk Factors

An array of reports highlighting the existence or, in some cases, the absence of disparities in the traditional risk factors for vascular diseases between CEECs and WECs is available. Demographic characteristics are important factors that are associated with atherosclerosis. In this sense, while the entire European population shows shrinking and ageing, a marked divide is discernible in the long-term population trends of WECs and CEECs. WECs are experiencing a rising population due to a mild natural population increase and positive net migration from CEECs and non-EU countries. In contrast, in CEECs, a fast shrinking of the population is observed due to lower fertility rates and significant negative net migration. Emigration from these countries is mainly of the working-age population, driven by opportunity differentials (employment and education opportunities, wage levels), and impacts both the size and the age structure of these populations [8]. Although the old-age dependency (the ratio of those over 65 years of age to the working-age population) is currently higher in WECs than in CEECs due to higher life expectancy, the latter countries will rapidly reach old-age dependency ratios higher than those already considered problematic in comparatively rich WECs [9]. In this sense, in relation to demographic changes, the burden of vascular disease in CEECs is expected to rise sharply in the coming years. In addition to these demographic transitions, lifestyle and comorbidities contributing to vascular risk are also worth analysing.

There is a consensus that smoking represents the single largest avoidable health risk in Europe [10]. According to the European health interview survey data in 2014, daily tobacco use in Europe was high, ranging from 8.7% in Sweden to close to 27% in Bulgaria and in Hungary (18% on average in EU28). In comparison of WECs and CEECs, the daily use of tobacco is remarkably higher in CEECs (16% vs. 23%). Beyond the location of people, educational attainment and income are also influential in this regard. The sex differences (predominance of men among smokers) and the proportion of heavy smokers were also higher; the quit ratio was lower in CEECs [11].

In addition, the loss of life attributable to premature death caused by tobacco use is much higher in CEECs than in WECs. This was also observed for rate of deaths caused by CVD and cancer. Moreover, the highest value of disabled life burden (YDL), expressed in years that was attributable to smoking-related CVD across the globe was found in Eastern Europe in 2016. In Central Europe, a small reduction was seen in men during the same period. However, the values of YDL were lower in WECs in both sexes [12]. In a recent publication, based on an age–period–cohort model, the age-specific and age-standardised smoking-attributable mortality fractions showed a later and higher peak in CEECs, compared with WECs. However, based on the projected values up to 2100, these numbers are predicted to converge [13]. In smokers, a healthy diet was associated with better CVD mortality. This effect was more pronounced in CEECs than in WECs, indicating disparities in nutritional habits [14].

Elevated cholesterol level is a well-established risk factor for atherosclerotic vascular diseases and requires effective lifestyle modification, changes in diet, and aggressive lipid-lowering therapy [15]. Based on a pooled analysis of 1127 population-based studies that measured blood lipids in 102.6 million individuals aged 18 years and older, that aimed to estimate trends in cholesterol from 1980 to 2018, the global epicentre of non-optimal cholesterol level drifted from WECs and CEECs to Asia and the Pacific. A similar decline in cholesterol level was seen in WECs and CEECs. The decrease was the largest in Northwestern Europe. This tendency was thought to be related to changes in diet and the implementation of effective statin therapy [16]. However, in the results of the Centralized Pan-Regional Surveys on the Undertreatment of Hypercholesterolaemia (CEPHEUS), conducted in 29 countries globally, based on data from 16,973 patients from Europe, a difference was found between WECs and CEECs in the proportion of patients attaining the low-density lipoprotein (LDL) cholesterol goal (NCEP ACT III guideline). While this proportion was shown to be 56.6% in WECs, in CEECs it was only 26%. The difference was especially marked for patients living with high and extremely high cardiovascular risk [17]. These results were supported by an observational study that recruited 1244 patients from CEECs with high and extremely high cardiovascular risk, which revealed that less than one-quarter of high-risk cardiovascular patients and less than half of extremely high-risk cardiovascular patients achieved their LDL targets. Additionally, less than 15% of patients with familial hypercholesterolaemia reached these targets. According to the authors’ interpretation, beyond statin discontinuation/poor adherence, financial/reimbursement issues may play an additive role in these findings. Only half of these patients (53%) were taking high-intensity statin therapy, and only 13% were receiving statin + ezetimibe combinations [18].

Based on the data from the International Diabetes Federation, in 2019, the age-adjusted prevalence of diabetes in Europe was 6.3% on average, which ranged from 4.9% to 9.2% across the different countries, emphasising a marked variation. In CEECs and WECs, the prevalence of diabetes was 7% vs. 9%, respectively [19]. In another pooled analysis of 751 population-based studies, focusing on worldwide trends in diabetes since 1980, the age-adjusted diabetes prevalence showed almost no change in women in WECs. In men, however, a small increase was detectable throughout Europe [20].

An analysis of the worldwide trends in blood pressure from 1975 to 2015 (1479 studies that had measured the blood pressures of 19.1 million adults) showed that the highest worldwide blood pressure levels have shifted from high-income countries to low-income countries in South Asia and sub-Saharan Africa, while blood pressure has been persistently high in the CEEC region. Central and Eastern Europe, sub-Saharan Africa, and South Asia had the highest mean blood pressures in 2015 [21]. Not only the high blood pressure load, but also the between-visit variations in blood pressure may contribute to the high cardiovascular risk profile in CEECs [22].

Accumulating observations support that the socioeconomic status of the individual and their environment profoundly influence the cardiovascular prognosis [23,24]. Thus, the complex interplay of biological (genetic), behavioural (diet, physical activity, smoking, alcohol consumption, drug addiction), material (poverty, unemployment, poor housing, lack of access to and availability of health care), and psychosocial (stress, depression) determinants contribute throughout the ‘life course’ to the CVD risk [24].

In these dimensions, several findings show disparities between CEECs and WECs. In the study of Health, Alcohol and Psychological factors in Eastern Europe (HAPIEE), six psychosocial and socioeconomic factors (unemployment, low material amenities, education, depression, being single, infrequent contacts with friends or relatives) were associated with increased risk of death from CVD [25]. The differences in subjective well-being in CEECs compared with other countries, including WECs, referred to as the happiness gap, are well documented in many surveys from the early 1990s to 2014 (the Pew Global Attitudes Survey, the Eurobarometer, The European Values Study, The Life in Transition Survey) [26]. In an assessment of the physical and emotional aspects of heart disease-specific health-related quality of life (HRQL), a study that recruited 6384 patients with ischemic heart disease from 22 countries, a markedly lower level of HRQL was detected in CEECs compared with WECs [27]. These results are thought to be attributed to a perception of low income, weak overall business or regulatory environments, corruption and poor governance [26]. Another analysis, using retrospective data from the Survey of Health, Ageing, and Retirement in Europe (SHARE) [28], drew attention to the importance of the long-lasting impacts of psychosocial stress and financial hardship during adulthood on health over the life course. According to these results, the effect of stressful periods, financial hardships, and job loss occurring around the transition (1987–1993) in CEECs was associated with the subjective and objective measures of health in 2017. However, the consequences of hardships were not found to be specific to the transition; the health implications of these difficulties seemed to be similar to those of other shocks possibly unrelated to the transition, such as economic crisis [29].

In CEECs, health system outcomes are thought to be influenced by a wide range of economic, political, social, and systemic factors that may contribute to the overall risk of vascular conditions [30].

### 3.2. Structure, Processes, and Outcomes of Vascular Care in CEECs and WECs

Using Donabedian’s framework, which defines quality in health care as a function of three domains (structure, process, and outcome) [31], further differences can be identified in a comparison of CEECs and WECs. However, available data are rarely specific for vascular care, indicating a marked gap requiring exploration.

### 3.3. Structure Disparities

Regarding structure (that is, the settings in which care is provided), according to a recent report by the European Commission, marked inequalities in access to health care across Europe are discernible. This simplified concept of access includes additional interlinked dimensions, such as (a) population coverage, (b) affordability of health care (cost-sharing), (c) basket of care, and (d) availability of health care (distance, waiting times) [32].

In the comparison of CEECs with WECs, a significant difference can be seen in the public health expenditure of the societies. The CEEC region shows a continuous lag in health expenditure, as indicated by an analysis of the period from 1994 to 2014 [33]. In 2017, the average health expenditure in percentage of gross domestic product was 6.8% for CEECs and 9.4% for WECs. The same divide expressed in purchasing power standards (PPS) was 1515 and 3107, respectively. This measure, which is an artificial currency unit, adjusts for differences in price levels between the EU Member States. Theoretically, one PPS can buy the same amount of goods and services in each country [34]. However, it must be added that the differences in health expenditure between WECs and CEECs cannot be reduced to public spending. Furthermore, the market price of medical devices and drugs provided to patients with vascular diseases are likely different between countries, what was only marginally reflected by simplified price models. The evident lag in public spending on health in CEECs entails a shift to the patients’ households in the form of out-of-pocket paying. This includes cost sharing (co-payment in a benefit package, direct payments for privately purchased healthcare), and even informal paying that leads to inequality in access to the service [35]. In addition to the complex scenario of lower spending and the higher financial request from households, leading to higher variations in access, CEECs seem to attract increasing interest from corporate and financial investors in medical technology. This trend is likely due to demographic changes, rising disposable individual income, and market fragmentation. The risk of investments in CEECs is directly linked to government policy and funding, corruption, and ownership law [36]. However, it also represents a risk regarding overuse of service and overtreatment that is not in harmony with the real medical needs [37].

No validly comparable data are found on spending on vascular care in CEECs and WECs. However, many other areas of the healthcare service show an East–West divide (worse in CEECs), including lack of availability of health professionals due to reduced numbers of professionals entering the labour market, or medical professionals leaving to work in more attractive areas (particularly urban centres) or to work abroad or in the private or non-contracted sector, regional disparities in health services, underfunding of the health system, and staff shortages in the publicly funded sector [32]. No in-depth analysis of the migration of vascular professionals in Europe is available; however, a shortage of vascular surgeons is reported in the UK and in the US, which shows much more capability to maintain the workforce in contrast to CEECs [38,39]. As a consequence, the lack of vascular specialists in CEECs is assumed to be an influential factor impeding the provision of vascular care in these countries.

In this sense, the free mobility of healthcare professionals in Europe seems to be at odds with the WHO global code of practice on the international recruitment of health personnel, which emphasises, as a guiding principle, that migration should equitably strengthen health systems (Art 3.2). There is more evidence for the drawbacks of mobility for the healthcare systems in the source countries than for its merits, but both the destinations and the sources experience positive and negative effects [40]. This complexity represents a challenge for CEECs: how can we implement different policy options to mitigate the unwanted effects and strengthen the positive effects of free workforce mobility [41]? To date, no exploratory analysis is available on this topic in the vascular field.

### 3.4. Processes

Process indicators reflect how vascular service is delivered and what is being done to improve the outcomes. Several features of these metrics support their use in service quality assessment [42]. The comparison of process indicators between WECs and CEECs is seriously hampered by the availability of data. Even in the frame of the VASCUNET collaboration, which aims to provide comparable scientific data on everyday clinical practice across Europe, only a few CEECs were involved (Hungary, Slovakia, Serbia, and Russia). While Hungary was among the founding members of the VASCUNET, others joined the collaboration during the past years. Often, data were limited to one specific issue. In addition, some country-specific publications are also accessible.

Our report from Hungary, which included a rough comparison, revealed that the volume of lower limb revascularisation procedures per capita is a fraction (50%) of the similarly defined procedures performed in the more developed WECs. In addition, according to the principle of the endovascular-first strategy, the characteristic predominance of endovascular procedures took place ten years later in Hungary compared with WECs [43]. This lag was supported by a VASCUNET report showing that the proportion of endovascular peripheral vascular operations, compared to open procedures, was the lowest in Russia (24%), while the proportion in WECs ranged from 57% to 88%. Additionally, it was shown that the proportion of patients presenting with intermittent claudication vs. chronic limb-threatening ischemia was the highest in Russia (69%), while the average proportion in WECs was 37% [44].

However, marked heterogeneity can be seen on this regards in WECs. In addition, as previous VASCUNET reports revealed, the average rate of popliteal artery aneurysm repairs per capita was lower in Hungary and in Serbia than in WECs (5.4 vs. 7.4 per 10^6^, respectively) [45]. Regarding abdominal aortic aneurysm repairs, as the sole representative of CEECs, Hungary, compared to seven WECs, showed a lower proportion of endovascular procedures in a relatively younger population (in cases of intact aneurysm, 17.5% vs. 35.3%, respectively; in cases of ruptured aneurysm, 4.8% vs. 17.5%, respectively). The comorbidity burden of patients was also higher in Hungary. However, it must be added that, in contrast to WECs, the rate of registry coverage was lower in Hungary [46].

In conclusion, a comprehensive comparison of data on process indicators of peripheral vascular care in WECs and CEECs is not feasible due to the scarcity of information. However, available data show a palpable disparity, and these data can be considered a signal of unwarranted variation between countries with room for improvement in CEECs. This was especially emphasised by the number of vascular procedures per capita and technological development.

### 3.5. Outcomes

Although process indicators seem more appropriate to assess the specific quality of care, outcome measures serve as hypothesis-generating tools that may contribute to the better understanding of complexity of vascular care.

One of the most solid outcome measures of CVD is the mortality associated with it. While CVD mortality shows a remarkable decline in Europe, in CEECs it is notably higher. Not only the CVD death rates are higher, but they occur in younger ages. Beneficial trends in WECs are thought to be the consequences of combined effects of lifestyle changes, public policy, and new, more effective medical treatments for CVD [47]. This association was also confirmed in Czech Republic representing CEECs [48].

While an abundance of data can be found on the mortality of patients with peripheral arterial occlusive disease [49], lower limb revascularisations [50], abdominal aortic aneurysm [51], or carotid artery disease [52] in WECs, reports from CEECs, especially large-scale population studies, are rare [53,54].

**Figure 1:**
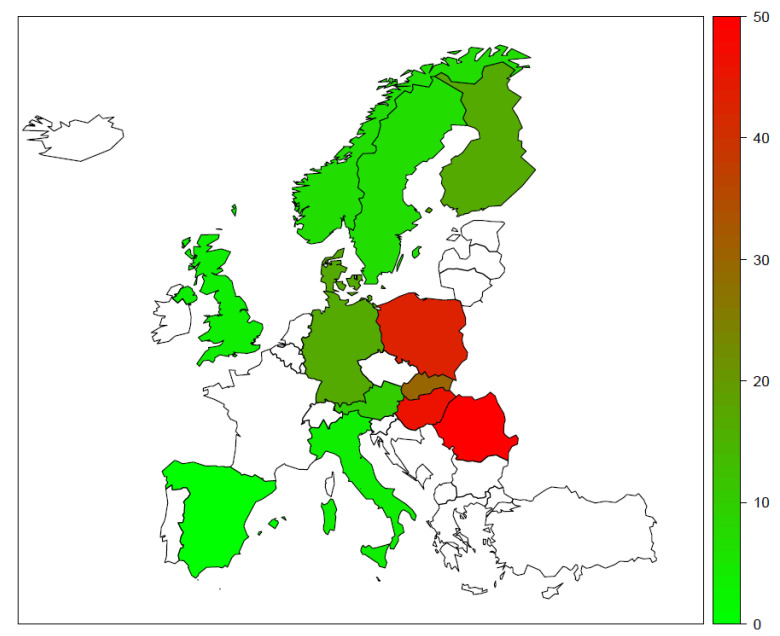
Rough estimates of major lower limb amputations crude incidences (per 10^5^) across Europe. In countries where data only for diabetic patients were available, incidence rates were doubled.

Lower extremity amputations serve as an essential distal outcome measure of vascular care. While several reports on and comprehensive analyses of amputation statistics from the WECs have emerged in the past decade, similar data from the CEECs are sparse [55]. Moreover, the differences between the methodological approaches allow only a rough comparison of data. If we use the published data and recalculate them to have the formula of crude incidence, it emerges that, on average, the CEECs have values close to or above 30 per 10^5^. These values are 42 per 10^5^ in Hungary [43,56], 29 per 10^5^ in Slovakia [55], 38 per 10^5^ in Poland [57], and 43 per 10^5^ in Romania [58]. In contrast, in WECs, the major amputation incidence data show values below 20 per 10^5^ [55,59] (Figure 1). This East–West divide was also discernible in unified Germany when Western and Eastern states were compared [60]. One report from CEECs showed that, as with Western countries, a small decline in amputations became apparent over a longer period, but the starting time was found to be ten years later compared to WECs [43]. Based on these data, an East–West divide in amputation outcomes is discernible [61].

## 4. Discussion

In this comprehensive narrative review, we attempted to explore whether an East–West divide is discernible in the peripheral vascular field across Europe. We analysed available data on vascular risk factors, vascular care settings (structure, processes), and outcomes. Considering data collection, it became evident that a meaningful comparison is significantly hindered by the lack or paucity of robust scientific data on vascular care from CEECs. Although the CEECs successfully adopted cohesion policy instruments of the EU in many fields, they are still somewhat behind in the development of science and innovation. As a consequence, a lag in scientific research and development and, specifically, scientific reporting from CEECs challenged our efforts. The disparity in scientific publication activity, measured by any metrics (number of articles, citations, expenditure per article, acceptance in the most respected scientific journals in the Web of Science), accounts for a spatial unbalance of data [62]. Underrepresentation of CEEC population data in vascular guidelines may distort the global validity of recommendations. Disparities in research may call upon an action to support regional diversity of cardiovascular scientific efforts [63]. Moreover, not only is there a lack of available scientific data on health care characteristics of CEECs, but the use of these data to support the health policy decisions is also limited. In that sense, in the frame of health technology assessment (HTA), which covers a broad spectrum of analysis of care (efficacy, safety, feasibility, cost, and cost-effectiveness, as well as social, economic, and ethical consequences) [64], in CEECs, the HTA bodies focus mostly on pricing and reimbursement. Due to the lack of national input data, in-depth analysis is rare. Decisions are based on data from elsewhere and expert opinions. Instead of using HTA as a mere administrative procedure to fulfil (inter)national requirements, these countries need an adequate national analytical capacity to assess and appraise technologies in the context of local need and affordability [65].

Beyond the support and development of HTA in CEECs, the facilitation of scientific data gathering and sharing in this region seems to be essential to explore disparities. In this regard, research and reports on national data, as well as cross-European scientific collaborations using large data from administrative or clinical registries, are of primary importance [66,67].

While our exploratory analysis revealed a marked disparity on more aspects of vascular care between WECs and CEECs, more research and a systematic review of the available literature would be required. In addition, differences among CEECs, and time trends, also remain to be explored.

While the EU Member States have the main responsibility for health policy and the provision of health care, there are areas where addressing common challenges at the EU level may provide added value [68]. EU institutions, bodies, and agencies contribute to reduce health inequalities through an array of policies, programs, initiatives, and instruments that affect the socioeconomic determinants of health. These contributions involve the European Council, the Social Protection Committee, the European Commission, the European Funds, the European Parliament, the European Commission Statistical Office, and the European Consultative Committees [69]. In this field, however, peripheral vascular diseases are underrepresented, which calls for action from the scientific vascular communities. Effort in supporting convergence in the health status of patients with peripheral arterial disease in WECs and CEECs may represent a common goal across Europe. It would be in harmony with the political aim, commitment, and values of the European Union.

## 5. Conclusions

In this comprehensive narrative review of the literature, a marked disparity between Western vs. Central and Eastern European Countries was found in terms of healthcare provided to patients with cardiovascular disease, emphasising an unwarranted variation. While there are data suggesting that Central and Eastern European Countries are underprivileged in different domains of vascular care, it also became evident that there is a distinct paucity of research data generated in those countries.

## Figures and Tables

**Table 1:** Domains of disparities in peripheral vascular care between Western European countries versus Central and Eastern European countries.

Domain of Differences	Examples
Vascular risk factors	Demography, tobacco use, hyperlipidaemia, diabetes, socioeconomic status, environment
Structure of vascular care	Health expenditure, availability of vascular specialists
Processes of vascular care	Volume of vascular procedures, availability of new technologies
Outcomes of vascular care	Mortality, lower limb amputations
Availability of scientific data	Backwardness in research and development, organisation of health technology assessment

## Data Availability

Not applicable.
